# Diversity Patterns and Assembly Mechanisms of Heteroptera Communities Under Environmental Change

**DOI:** 10.1002/ece3.72009

**Published:** 2025-09-17

**Authors:** Fuyuan Ta, Weina Wang, Hongru Yue, Namuriga, Husile, Junlan Li, Ning Wang

**Affiliations:** ^1^ College of Life Sciences Inner Mongolia University Hohhot China; ^2^ Institute of Grassland Research Chinese Academy of Agricultural Sciences Hohhot China; ^3^ Chifeng Zhaowuda Senior High School Chifeng China; ^4^ Xilingol League Grassland Workstation Xilinhot China; ^5^ Hulunbuir Forestry and Grassland Development Center Hulunbuir China

**Keywords:** community assembly mechanism, community stability, diversity patterns, Heteroptera

## Abstract

The species richness of Heteroptera communities is closely linked to the diversity of local arthropods, suggesting that they can serve as reliable indicators of local biodiversity levels. This study focused on heteropteran communities in the Daqing Mountain National Nature Reserve, elucidating the diversity patterns and assembly mechanisms of heteropteran, as well as the primary driving factors. A survey of the heteropteran communities was conducted from May to September 2022 using sweep‐net sampling. The results demonstrate that heteropteran species diversity increases along elevational gradients, and a few ecological variables serve as significant drivers of species richness. Ecological modeling revealed that deterministic processes predominantly govern heteropteran community assembly across the nature reserve, while stochastic processes play a contingent role in insect community assembly across elevational gradients. Based on these findings, we screened relevant ecological variables and constructed a random forest model to estimate the spatial distribution patterns of heteropteran species richness within the nature reserve, revealing a distinct east‐high‐west‐low spatial distribution pattern. Further analysis of community stability demonstrated that heteropteran species diversity serves as the primary determinant of community stability. Collectively, this study provides novel insights into the impacts of environmental change on heteropteran population stability and aggregation dynamics within the Daqing Mountain Nature Reserve, while also informing the development of biodiversity conservation measures and sustainable reserve management strategies.

## Graphical abstract

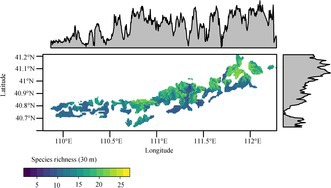

## 1 Introduction

Biodiversity is the foundation of human survival and development and serves as a key indicator for assessing the ecological sustainability of a region (Christensen et al. 1996). Insects are an excellent model for studying diversity patterns due to their vast population sizes, high species richness, broad distribution, strong adaptability, and small body size, which enables them to occupy diverse habitats even at fine spatial scales. Therefore, they play a vital role in biodiversity conservation and research (Chen, Huang, et al. 2019). Nature reserves provide abundant food resources and suitable shelters for insect growth and development, contributing to biodiversity maintenance and species distribution patterns (Swart et al. 2021). Among insect groups, Heteropteran insects, commonly referred to as “true bugs,” are considered to have a relatively lower degree of evolutionary advancement. This diverse group encompasses species with considerable variation in body size, dispersal ability, host specificity, and trophic level (Dolling 1991). Both larvae and adults typically inhabit the same habitat, displaying pronounced phylogenetic conservatism (Vialatte et al. 2010). The species richness of Heteroptera communities is closely linked to the diversity of local arthropods, suggesting that they can serve as reliable indicators of local biodiversity levels (Duelli and Obrist 1998). Furthermore, their sensitivity to environmental changes makes them excellent indicators for monitoring environmental shifts (Attignon 2004; Gerber et al. 2008).

The distribution pattern of insect diversity and the mechanism of its maintenance are hot issues in the field of insect ecology (Adams et al. 2020). The short generation times of insects and the established standardized collection and identification methods make them suitable for ecological monitoring, and they are highly responsive indicators of environmental heterogeneity and change (McIntyre 2001). The vegetation structure, spatial location, and the degree of surrounding landscape fragmentation all influence the structure of local insect communities (Filgueiras et al. 2019). Differences in vegetation structure directly affect insect breeding sites, habitats, food sources, and refuges, and consequently impact insect diversity (Fahrig et al. 2011). Some studies have indicated that plant diversity is a key factor affecting the species richness, abundance, and composition of true bugs across different habitat types (Chen et al. 2010). The higher the vegetation richness in a habitat, the greater the Heteroptera diversity (Gessé et al. 2014; Birkhofer et al. 2017). Comparisons of the impact of land use types and elevation gradients on insect community characteristics have revealed that insect species richness is not significantly related to land use types; however, there is a significant relationship with elevation, indicating that spatial location is important for insect diversity (Birkhofer et al. 2017). Habitat connectivity and microhabitat diversity are also of great importance for the conservation of insect diversity (Hirao et al. 2013). However, research has only explored the responses of insects to habitat structural changes at small scales. Therefore, there is a need for research on the structural changes of insect communities at the regional scale to improve understanding of insect responses to environmental changes, as well as the effects of ecological factors, such as vegetation, topography, and climate, on insect communities (Antão et al. 2019).

Research frameworks integrating niche‐based theory and neutral theory offer insights into community assembly mechanisms (Grossiord et al. 2014; Ma et al. 2018). However, the relative contributions of deterministic processes (homogeneous or heterogeneous selection) and stochastic processes (dispersal limitation and homogenizing dispersal) vary significantly across taxa and environments (Liu et al. 2021). For instance, deterministic processes dominate plant community assembly in temperate grasslands, whereas stochastic processes primarily govern soil fungal communities and Eurasian steppe microbial assemblages (Xu et al. 2022). Ferrenberg et al. (2016) revealed that environmental factors such as vegetation cover exert stronger influences on aboveground arthropod communities due to their distinct ecology and dispersal capacity, whereas stochastic processes predominantly govern belowground communities. Stochastic processes dominate the assembly of butterfly communities in subtropical montane nature reserves and coleopteran communities in oil palm plantations. In contrast, deterministic processes drive community assembly across various land‐use types at multiple spatial scales (Edwards et al. 2021; Wei et al. 2024). These mechanistic divergences highlight the necessity for systematic investigations tailored to distinct ecosystems and biological groups to uncover universal principles underlying the maintenance of community stability under environmental change.

The Yinshan Mountain Range serves as a vital natural and ecological boundary in northern China, and the Daqing Mountain National Nature Reserve is located in the middle section of the range. This reserve is one of the most ecologically diverse areas and plays a crucial role in soil and water conservation, windbreak and sand fixation, carbon sequestration, and oxygen release. Additionally, it functions as a key water recharge area for the upper and middle reaches of the Yellow River, significantly contributing to the regulation of water balance and supply in northern China (Li 2014). Due to its unique geographical position and ecological characteristics, the Daqing Mountain Nature Reserve is highly sensitive to global climate change and encompasses a relatively complete range of ecosystems, including forests, grasslands, and wetlands. Moreover, it serves as a significant biodiversity distribution area in northern China (Yin and Wang 2021). While biodiversity research within nature reserves has long focused on vertebrates and plants, we are still far from understanding the general diversity patterns of insects, which are the most species‐rich taxa in the animal kingdom and play an important role in ecosystems and human life (Beck et al. 2017). Biological communities form dynamic systems of varying stability through complex species interactions (Barberán et al. 2012). While a strong correlation between stability and complexity has been established, the regulatory role of complexity in governing stability under environmental changes remains unclear (Yuan et al. 2021; Landi et al. 2018). Taxonomic diversity (through species‐specific responses to environmental fluctuations) may theoretically enhance community stability, yet recent studies demonstrate that highly diverse communities do not necessarily exhibit greater resilience to disturbances; instead, increased diversity may amplify vulnerability due to heightened environmental stressors affecting more species (Li 2014b).

This study conducted a comprehensive survey of Heteroptera in the typical temperate mountainous area of the Daqing Mountain National Nature Reserve in Inner Mongolia, China, examining their community diversity, distribution, and assembly mechanisms. Based on these data, this study aimed primarily to address the following questions: (a) What is the pattern of heteropteran diversity, and what are the best environmental predictors? (b) What is the relative contribution of deterministic versus stochastic processes in governing heteropteran community assembly? (c) How do complexity and diversity affect stability under environmental change scenarios? The study will provide a comprehensive understanding of Heteroptera diversity patterns within protected areas, delivering invaluable data to inform targeted conservation measures and sustainable management strategies based on the key environmental drivers influencing heteropteran communities and their assembly processes across altitudinal gradients.

## 2 Materials and Methods

### 2.1 Study Area and Heteroptera Insects Collection

The Daqing Mountain National Nature Reserve, located in central Inner Mongolia, China, is part of the Yinshan Mountains. It spans an elevation from 1050 to 2374 m and covers a total area of 38.89 km^2^, extending across Hohhot, Baotou, and Ulanqab. The reserve lies in a temperate semi‐arid zone characterized by a continental climate, with an average annual precipitation of 400 mm, an annual evaporation of 1800 mm, an average annual temperature of 5.9°C, and an annual sunshine duration of 2976.5 h. It experiences an annual accumulated temperature of 2800°C above 10°C and a frost‐free period ranging from 90 to 180 days. The predominant soil types in the reserve are leached gray‐brown soil, skeletal soil, gray‐brown soil, and grass‐shrub gray‐brown soil. The Daqingshan Nature Reserve is a comprehensive mountainous forest‐type protected area, primarily consisting of mountain forests, shrublands, and mountain grasslands. It also serves as an important water source conservation area (Li 2014).

**FIGURE 1:**
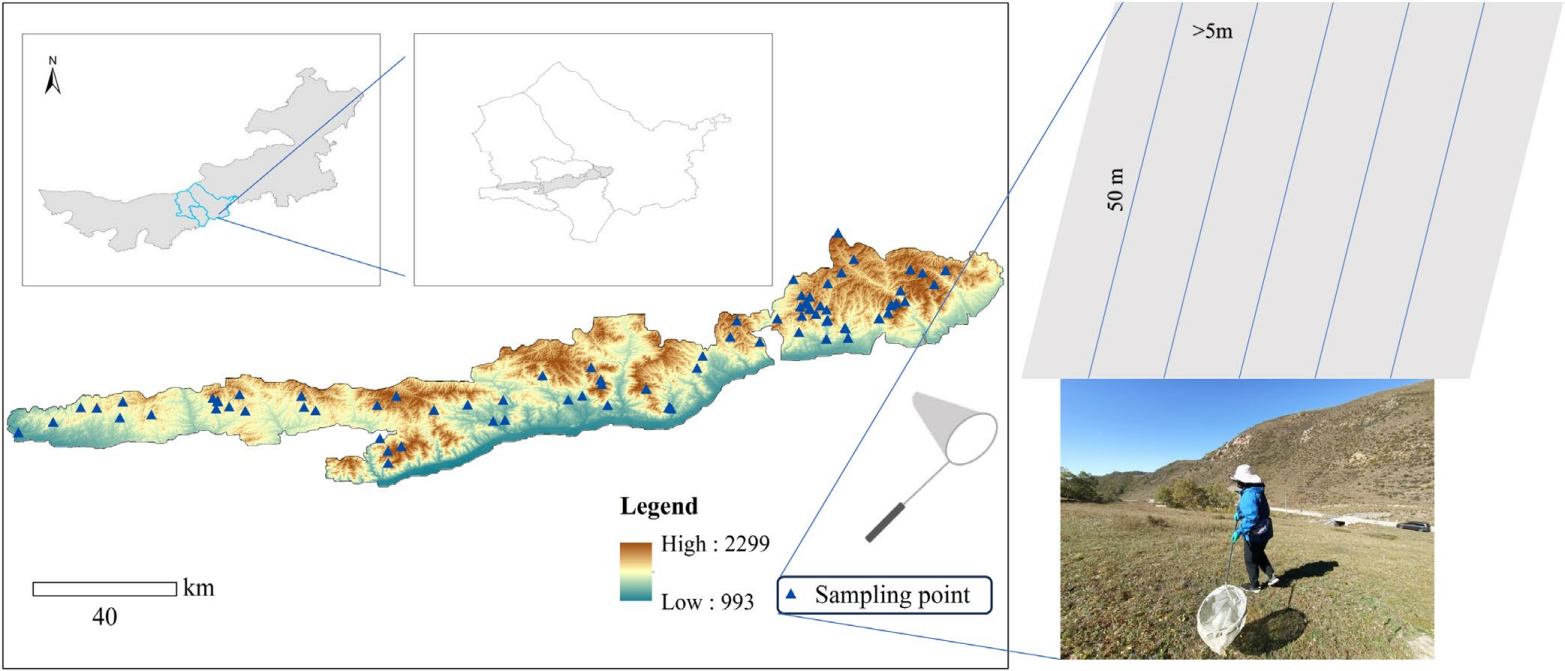
The location of the experimental zone and sample plot distribution. DEM, digital elevation model.

A survey was conducted that covered a total of 110 sample points (Figure 1). The Heteroptera individuals were collected from May to September 2022. Within the sampling points, five parallel lines were selected (approximately 50 m in length, with a distance greater than 5 m between each pair of parallel lines). Between 09:00 and 12:00 on sunny and windless days, an insect net was held with the hoop positioned in the vegetation canopy and the opening facing vertically to the ground. The net was swung 180°, and one net sweep involved one sweep from left to right and back. While walking along each transect, 50 net sweeps were performed, totaling 250 sweeps per sampling plot, including five vertical sweeps every 5 m and five horizontal sweeps every 80 cm (Schaffers et al. 2008). The collected insects were stored in Eppendorf tubes containing 75% alcohol, and the collection time, location, elevation, and geographic coordinates were recorded. The interval between each sampling event was more than 20 days.

Species identification of all the collected specimens was performed using the Nexcope NSZ818 stereomicroscope, except for larvae that could not be classified due to not fitting any of the taxonomic descriptions (Malipatil 1978). For species with morphological ambiguities, identification was confirmed through dissection of the external genitalia. All specimens were re‐examined and authenticated by taxonomic experts before being deposited in a −20°C freezer at Inner Mongolia University, Hohhot, Inner Mongolia, China.

### 2.2 Environmental Variables

All the abiotic environmental variables in this study were obtained from various databases and remote sensing data sources. The normalized difference vegetation index (NDVI) was retrieved from the National Tibetan Plateau/Third Pole Environment Data Center (Gao et al. 2023; https://doi.org/10.11888/Terre.tpdc.300328, https://cstr.cn/18406.11.Terre.tpdc.300328) at a spatial resolution of 250 m. The soil data, including the soil pH, total nitrogen (TN), soil organic carbon (SOC), were sourced from the Harmonized World Soil Database version 2.0 at a spatial resolution of approximately 1 km (https://www.fao.org/soils‐portal/data‐hub/soil‐maps‐and‐databases/harmonized‐world‐soil‐database‐v20/zh/). Climate data were obtained from the National Tibetan Plateau/Third Pole Environment Data Center (https://data.tpdc.ac.cn/zh‐hans/data/faae7605‐a0f2‐4d18‐b28f‐5cee413766a2) at a spatial resolution of 1 km. The data included the monthly average precipitation (MAP, mm) and monthly average temperature (MAT, °C). The DEM were obtained from the elevation down loaded from the Geospatial Data Cloud (https://www.gscloud.cn/) with are solution of 30 m. The aforementioned variables, including the NDVI, MAP, MAT, SOC, TN, and pH, were extracted for the survey plots using the “Extract Multi Values to Points” tool in ArcGIS 10.2.

### 2.3 Diversity Patterns and the Importance of Environments

Prior to analyses, data from the five survey times were pooled at the plot level. The community diversity patterns of heteropterans were first analyzed using species richness (SR) and abundance as measures of taxonomic alpha diversity. To evaluate compositional dynamics in heteropteran communities, taxonomic beta diversities were quantified using pairwise Jaccard similarity coefficient indices. The derived diversity metrics were subsequently partitioned into nestedness and turnover components following established methodologies (BAS method) (Cardoso et al. 2014).

Variations in the community diversity of heteropteran in relation to elevation were assessed using Pearson's correlation coefficient, and the most important drivers of SR and abundance correlated with the seven environmental factors were identified using a random forest (RF) model. The “rfPermute” package (Archer 2024) was used to assess the impact of environmental factors on the SR and abundance of the heteropteran. The significance of these impacts was evaluated using permutation tests in the “rfPermute” package, which evaluated the relative contribution of the variables based on the mean standard error of prediction (% IncMSE) (Lie et al. 2012). The higher the mean standard error, the more significant the variable, and *p*‐values were calculated to determine significance.

### 2.4 Spatial Pattern of Heteropteran Species Richness

Estimated plot‐scale (100 m^2^) heteropteran species richness and mapped it over the Daqing Mountain National Nature Reserve with a grain of 30 m. Since there was a noticeable collinearity between the predictors in the ecological variables, we removed variables that contributed little to the predictions. Initially, we determined the ranking of the importance of the above predictor variables for heteropteran species richness in Section 2.3. Then, a backward stepwise regression method was applied to remove colinearity between variables with a variance inflation factor (VIF) less than 5, in order to maintain the best prediction accuracy for species richness (Table S1) (Genuer et al. 2015). Following these lines, we opted for five predictors: elevation, MAT, NDVI, pH, and SOC; they were finally selected as the final variables for estimating the species richness of heteropteran in the reserve. Finally, utilizing the terra package in R 4.4.0, we resampled the spatial resolutions of MAT, NDVI, pH, and SOC to a 30‐m resolution.

Then, we modeled the relationships between heteropteran species richness and selected predictors by using the RF model. The RF is a nonparametric and ensemble machine‐learning model that combines many decision trees to generate a prediction (Crisci et al. 2012). A set of hyperparameters was tuned in advance to optimize model performance (Bergstra and Bengio 2012). We first set the number of regression trees to 500. We then automatically controlled the minimal node size (from 4 to 10) and mtry (from 2 to 5) to the number of predictor variables to return the lowest RMSE with a 10‐fold random cross‐validation procedure (Table S2), using the train function in the R 4.4.0 package CARET (Kuhn 2024). Next, we used these best hyperparameters to refit the final model based on the R 4.4.0 package RANGER (Wright and Wiest 2024) (Zhao et al. 2024).

### 2.5 Assembly Mechanisms of Heteropteran Communities

Used the Sloan neutral community model (NCM) to evaluate the potential effects of neutral processes on the assembly of heteropteran communities in the nature reserve, which predicts that the rare taxa are more likely to be lost at different sites because of ecological drift, whereas taxa abundant in the metacommunity will be widespread because of their stronger dispersal ability among different sampling sites (Stamatakis 2014; Magurran 2005). The parameter *R* ^2^ indicates the goodness of fit to the neutral model. The estimated Nm represents the interaction between population size and migration rate in neutral community models, with higher values indicating a stronger influence of dispersal on community composition and a lower probability of dispersal limitation. Models were fitted using the “nlsLM” function from the “minpack.lm” package (Elzhov et al. 2016).

The relative importance of stochastic and deterministic processes in shaping heteropteran community assembly was assessed using the modified stochasticity ratio (MST) analysis, which is based on the assumption that the observed community composition deviates from that expected under a null model. The MST quantifies the proportion of community assembly variance explained by stochastic processes relative to deterministic processes. An MST value greater than 0.5 suggests that stochastic processes dominate, while a value less than 0.5 indicates deterministic processes are more influential (Liang et al. 2020). The above statistical analyses were performed in R 4.4.0 using the “tNST” function from the “NST” packages (Ning 2022).

### 2.6 The Effect of Complexity and Diversity on Stability Under Environmental Changes

The degree of complexity of heteropteran communities can be quantified using cohesion analysis by calculating the total abundance‐weighted pairwise correlations of every taxon in a given community, with cohesion values illustrating positive or negative species interactions and reflecting the degree of cooperative behavior and competitive interactions (Xun et al. 2021). In addition, the stability of heteropteran communities was evaluated by the average variation degree (AVD), which was calculated using the degree of deviation from the mean value of the relative abundance of normally distributed species at different elevation levels (Lefcheck 2016). AVD was fitted using the apply function from the vegan (Oksanen et al. 2013).

To further evaluate the direct, indirect, or both effects of complexity and diversity as possible mediators of heteropteran community stability, it employed Partial Least Squares Path Modeling (PLS‐PM). Within this conceptual framework, it is hypothesized that ecological variables primarily drive system stability by influencing community diversity and complexity. Subsequently, both community diversity and complexity of insect communities jointly regulate their stability through direct and indirect pathways. The variables included in the PLS‐PM analysis encompassed ecological variables (MAT, MAP, and NDVI), community diversity (SR and abundance), and community complexity (positive cohesion and negative cohesion). The Goodness of Fit (GoF) metric assesses the overall fit of the PLS‐PM model, with values ranging from 0 to 1, where higher values indicate better model performance. In this study, the GoF coefficient was approximately 0.41, suggesting a relatively good prediction of the model (Sanchez 2013). All the above statistical analyses were performed in R 4.4.0 using the plspm function from the “plspm” packages (Frederic Bertrand et al. 2024).

## 3 Results

### 3.1 Diversity Patterns and Their Ecological Drivers

**FIGURE 2:**
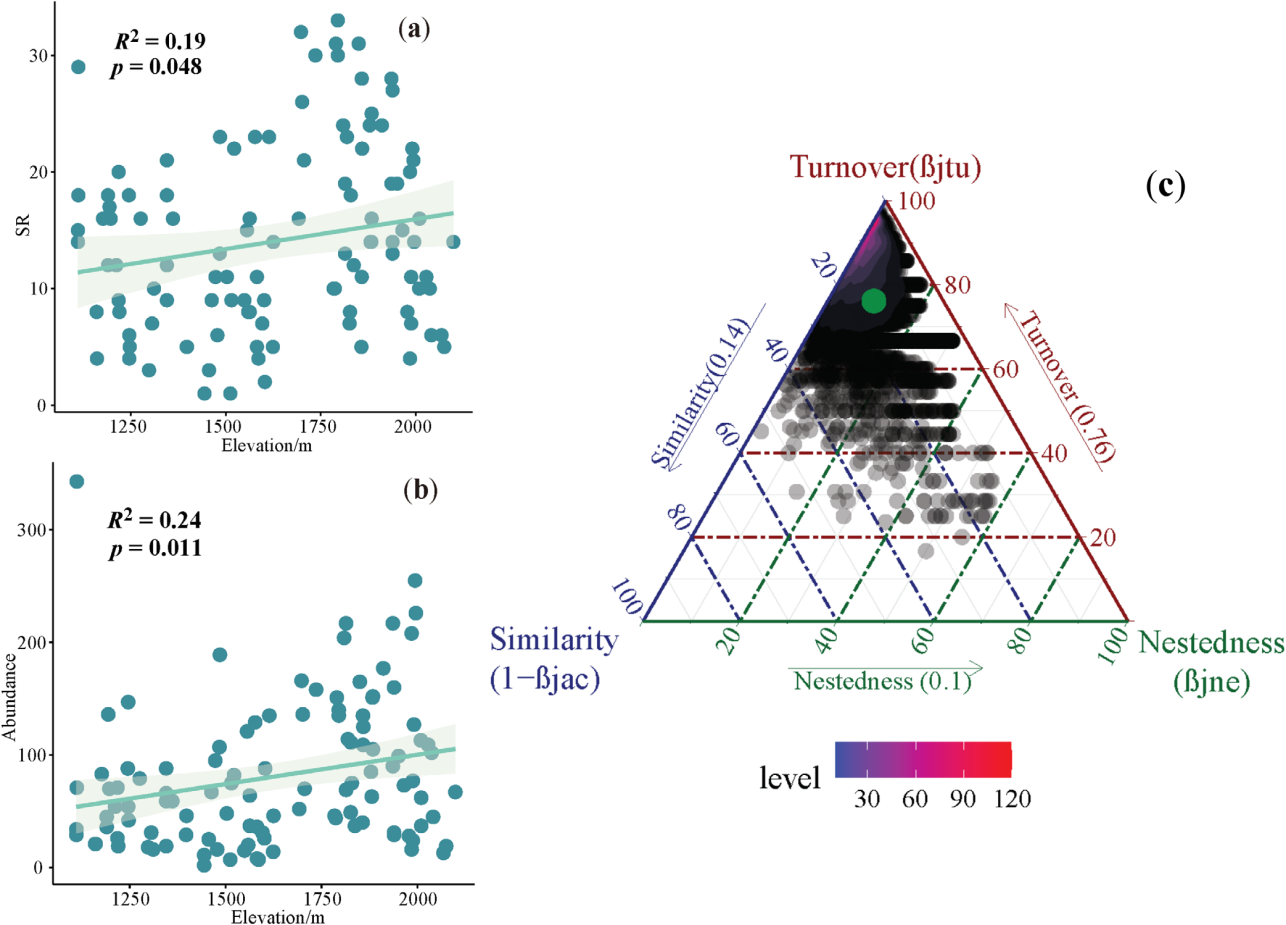
Variations in (a) species richness (SR), (b) abundance of heteropteran along altitude in the nature reserve; regression lines refer to the significant relationship between the two variables detected through linear models and shading areas associated with the lines represent the 95% confidence interval; (c) taxonomic beta diversity decomposition ternary diagram.

A total of 8909 Heteropteran insects were captured during the survey, belonging to 26 families, 103 genera, and 159 species (Table S3). Assessment of sampling coverage and species accumulation curves indicated that this survey found the vast majority of heteropteran species known to occur in the reserve (Table S4; Figure S1) the alpha diversity (SR and abundance) of the heteropteran community showed a significant positive correlation with altitude (Figure 2a,b, *p* < 0.05). The BAS analysis showed that the turnover component was the main contributing factor to the difference in the composition of the heteropteran community (76%), followed by the similarity component (14%), and the contribution of nestedness was the smallest, at 10% (10%) (Figure 2c).

**FIGURE 3:**
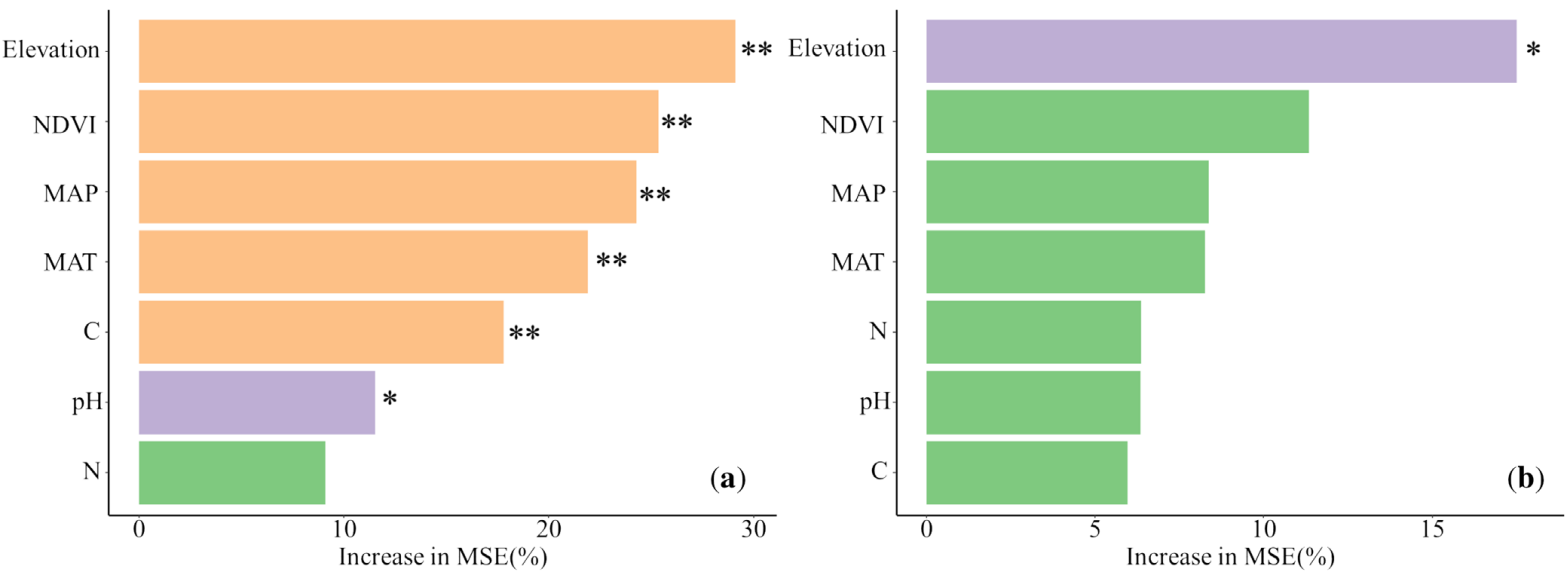
Importance of the seven environmental factors (analyzed using a random forest model) in driving (a) SR and (b) abundance. Significance levels: *p < 0.05, **p < 0.01.

A random forest model was established for the diversity of the heteropterans, and the quantitative analysis results (% IncMSE value) of the relationship between each indicator and environmental factor revealed that elevation was the primary factor affecting the diversity. Specifically, elevation, NDVI, MAP, MAT, and SOM exhibited highly significant effects on heteropteran species richness (Figure 3a, *p* < 0.01), with pH also demonstrating significant influence (Figure 3a, *p* < 0.05). In contrast, only elevation showed a significant association with heteropteran abundance (Figure 3b, *p* < 0.05).

### 3.2 Assembly Mechanisms of Heteropteran Communities

**FIGURE 4:**
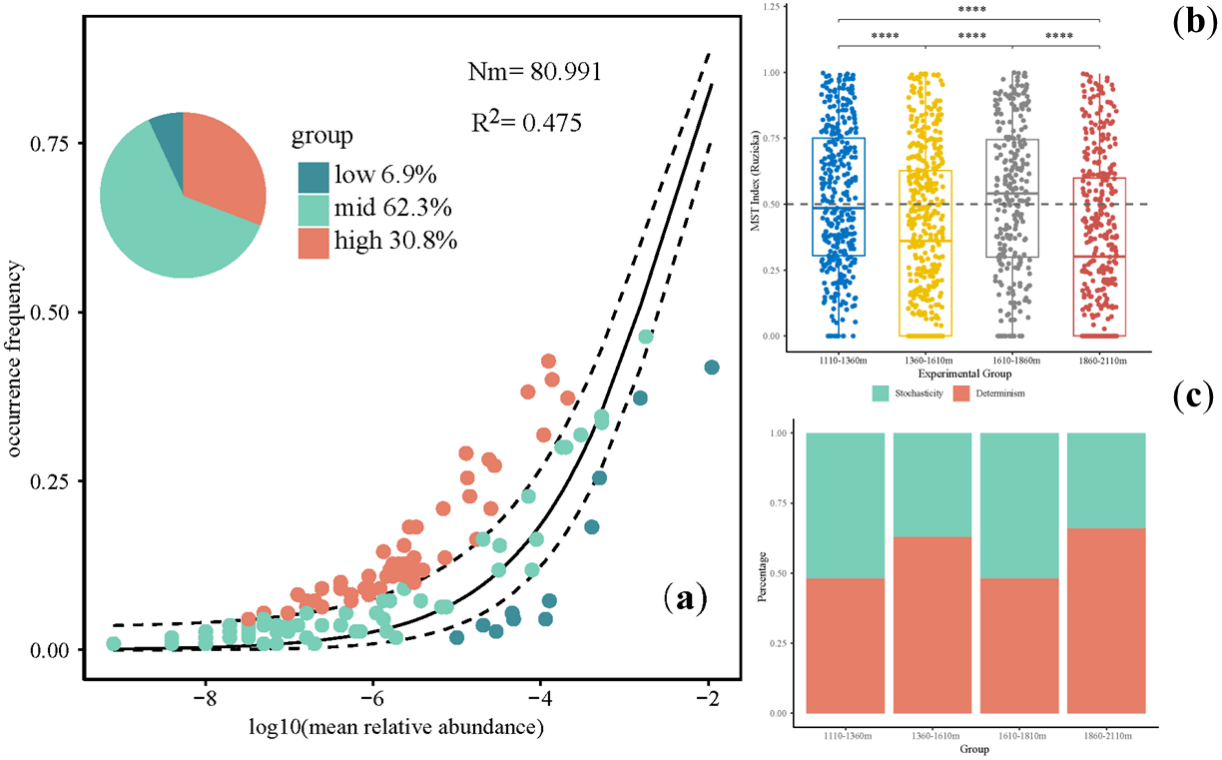
(a) The effects of random dispersal and ecological drift on the assembly of heteropteran communities through neutral model analysis. *R* ^2^ indicates the goodness of fit to the neutral model. Nm indicates the estimated migration rate. The solid black lines indicate the best fit to the neutral model and dashed black lines represent 95% confidence intervals around the model prediction. The relative abundance of the over‐represented (high), neutrally distributed (mid), and under‐represented species (low) in the heteropteran communities. (b) The significance of the difference in the values of modified stochasticity ratio index (MST) across different elevational gradients (1110–1360, 1360–1610, 1610–1860, and 1860–2110 m) tested by Wilcox. (c) The relative contribution of each ecological process to heteropteran community assembly in across different elevational gradients. Significance levels: ****p < 0.00001.

Overall, the NCM analyses showed that the frequency of Heteroptera had a moderate fit to the neutral model (*R* ^2^ = 0.48, *m* = 0.05, Nm = 80.99), the more restricted the movement of Heteroptera between communities. Furthermore, the ratios of relative abundances of neutrally distributed heteropterans were 62.3%, and the ratios of relative abundances of over‐represented heteropterans were 30.8% (Figure 4a). Thus, the underlying ecological processes could be inferred from the analyses of community turnover, and the relative importance of different ecological processes was assessed using null model analysis (Figure 4b,c). First, the results showed that highly significant differences (*p* < 0.001) were detected for the MST values for communities. In detail, it had highly significant differences between the 1110–1360, 1360–1610, and 1860–2110 m heteropteran communities; the 1360–1610 and 1610–1860 m communities, and the 1610–1860 and 1860–2110 m communities were also extremely significantly different (*p* < 0.001). Overall, distinct assembly mechanisms were observed across elevational gradients, with stochastic processes playing a dominant role specifically within the 1610–1860 m range. The heteropteran communities at 1110–1360 and 1610–1810 m were predominantly governed by stochastic processes, whereas deterministic processes dominated the assembly of communities at 1360–1610 and 1860–2110 m. Collectively, deterministic processes prevailed in shaping heteropteran communities across the Daqing Mountain Nature Reserve.

### 3.3 Spatial Pattern of Heteropteran Species Richness

**FIGURE 5:**
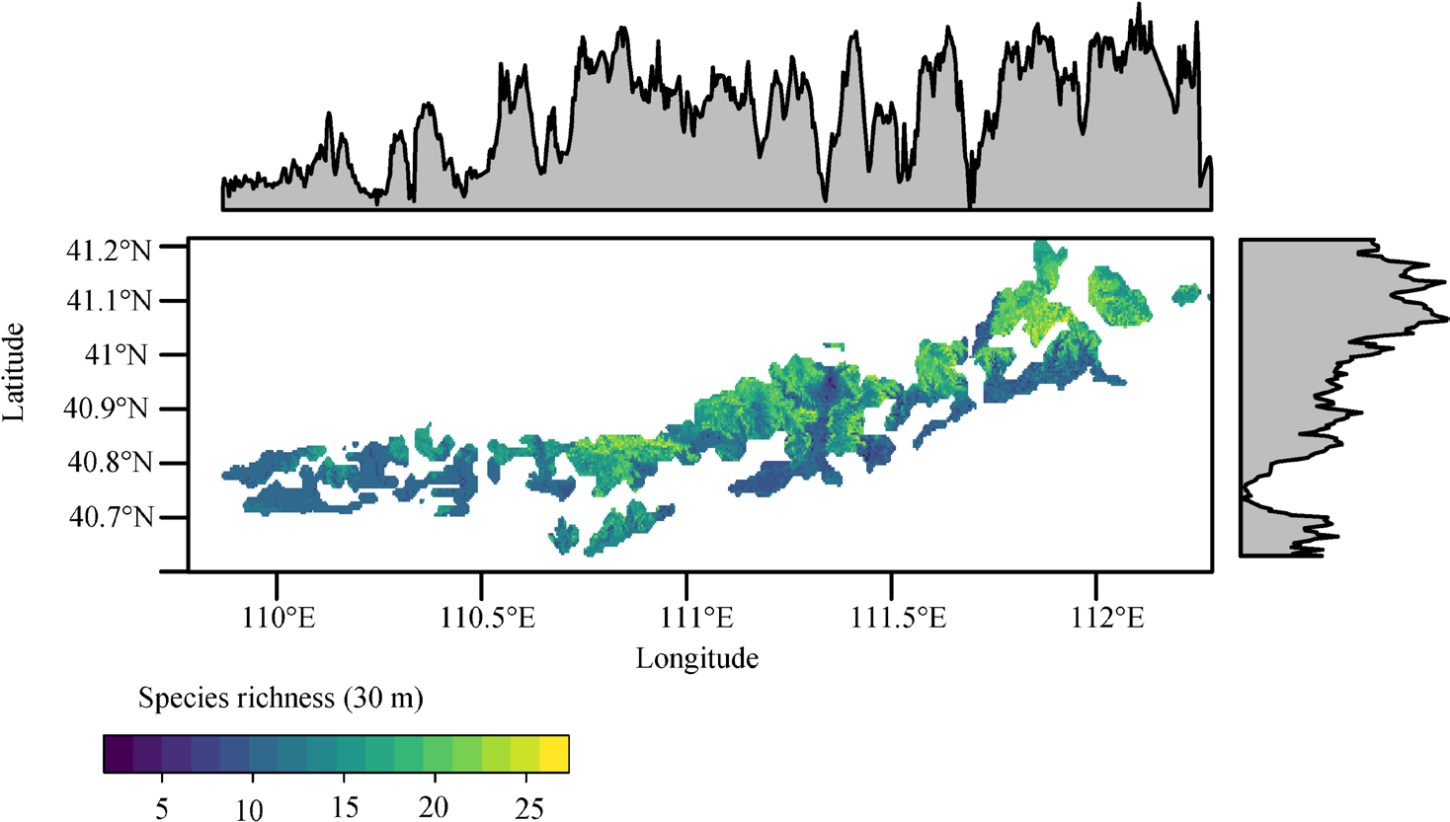
Estimated heteropteran species richness per 100 m^2^ in the nature reserve.

By combining all significant importance for heteropteran richness, macro‐environmental and remotely sensed variables, and using the ground‐measured species richness from the 110 sample sites, we predicted and mapped the heteropteran species richness of the nature reserve using a Random Forest (RF) model (Figure 5). The results showed that heteropteran species richness increased gradually from west to east along the longitude gradient across the nature reserve. Comparatively, heteropteran species richness exhibits pronounced spatial heterogeneity across the study region, with high‐richness areas demonstrating an island‐like distribution pattern. This phenomenon is likely attributable to the elevated habitat heterogeneity within the reserve. Consequently, conservation priorities should emphasize ecological protection of low‐richness zones and implement targeted management strategies to enhance landscape connectivity.

### 3.4 The Effect of Complexity and Diversity on Stability Under Environmental Changes

**FIGURE 6:**
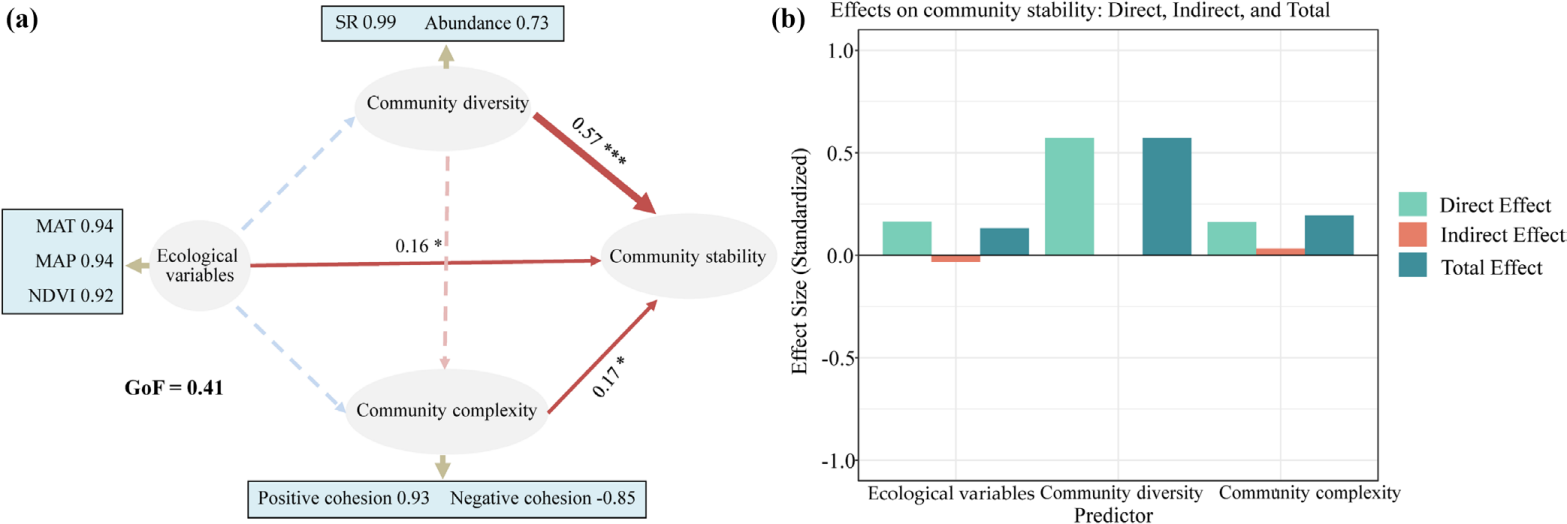
PLS‐PM shows the ecological variables, community diversity, and community complexity affecting heteropteran community stability (a) and the direct, indirect, and total effects of these variables based on standardized coefficients (b). Solid lines indicate significant positive (red) or negative (blue) relationships between variables (*p* < 0.05), and dotted lines indicate non‐significant relationships (*p* > 0.05). Arrow width is proportional to the strength of path coefficients. Numbers adjacent to measured variables represent the degree of correlation between the variable and its latent variable. With composite variables. Numbers adjacent to arrows represent the standardized path coefficients. Significance levels: *p < 0.05; ***p < 0.001.

In the Partial Least Squares Path Modeling (PLS‐PM) analyses, the ecological variables (MAT, MAP, and NDVI), community complexity, and community diversity have direct positive effects on heteropteran community stability in the nature reserve (Figure 6). Collectively, community diversity was identified as the principal driver of community stability, demonstrating direct regulatory effects, while abundance contributed secondly to stability, with SR contributing primarily. Both ecological variables and community complexity exhibited comparable magnitudes of influence on stability. Notably, ecological variables imposed weak negative indirect effects through conditional pathways, whereas community complexity enhanced stability via synergistic positive regulation through both direct and indirect mechanisms.

## 4 Discussion

### 4.1 Diversity Patterns

Existing studies have demonstrated that elevational gradients integrate variations in multiple environmental factors—including light, temperature, and humidity—significantly influencing insect community composition and diversity (Sanders and Rahbek 2012; Hodkinson 2005). Compared to longitude and latitude, altitude is recognized as the optimal parameter for investigating the impacts of global climate change on heteropteran diversity (Rasmann et al. 2014). In this study, altitude emerged as a critical environmental factor affecting heteropteran diversity in the protected area, with species richness (SR) and abundance exhibiting a linear increasing trend along the elevational gradient. However, distribution patterns of insect diversity across elevational gradients are complex and heterogeneous, demonstrating divergent trends in different regions, such as linear increases (Chatelain et al. 2018), multi‐peak patterns (Plant et al. 2019), linear declines (Laiolo et al. 2018; Zou et al. 2016), and U‐shaped distributions (Zhao et al. 2023). Consequently, under the synergistic effects of altitude and climatic variables, consensus on consistent elevational diversity patterns across regions remains elusive (Zou et al. 2016; Peters et al. 2016).

Heteropterans exhibit pronounced phylogenetic conservatism and have a strong influence on habitat changes (Vialatte et al. 2010; Gerber et al. 2008). Some studies have shown that differences in habitat types lead to changes in the community structure of different insect groups, and variations in the landscape structure of different habitat types can affect the communities. For example, the available food resources can influence the structure of insect communities (Pires et al. 2020). Consistent with previous studies partitioning beta diversity into turnover and nestedness components (Wang et al. 2018), the turnover component also emerged as the primary driver of compositional differences in heteropteran communities along the elevational gradient within the reserve. This finding suggests that high‐elevation communities are not merely subsets of low‐elevation assemblages and that both environmental filtering and dispersal limitation likely shape the species composition of heteropterans across distinct elevational zones (Müller et al. 2024).

### 4.2 Spatial Pattern of Heteropteran Species Richness

Insect diversity distribution patterns and their maintenance mechanisms represent a key research focus in insect ecology (Zhao et al. 2023). Environmental factors including altitude, climatic parameters, plant communities, and topography typically exert significant influences on insect community composition and diversity (Peters et al. 2016; Houghton et al. 2022). This study estimated the species richness of heteropteran insects in protected areas based on ecological factors demonstrating significant importance for their diversity, which provides crucial insights for elucidating heteropteran diversity distribution patterns. Utilizing high‐resolution remote sensing data, we successively generated 30‐m resolution species richness maps for heteropterans. However, the relative importance of remote sensing versus abiotic variables in predicting species richness exhibits scale dependency. At small spatial scales (< 1000 km), remote sensing variables demonstrate superior predictive capability compared to abiotic variables, as documented in previous literature (Zhao et al. 2024). Although sunny and windless periods were chosen for collecting the heteropteran insects in this study, numerous uncontrollable factors exist in the field, and occasionally gentle breezes may have affected the results of the sweep net sampling. Studies have shown that the species richness and Shannon index of Heteroptera can be best predicted by fine‐scale variables, such as local temperature, local relative humidity, and the presence of drought‐tolerant plants (Adams et al. 2020). Future studies should investigate multi‐scale modeling approaches to extend the coverage of in situ biodiversity measurements. Furthermore, establishing more refined quadrat surveys could enable comparative validation between field‐measured insect diversity and model‐estimated species richness.

In this study, the observed east‐high‐west‐low distribution pattern of heteropteran species may be attributed to multiple factors, including vegetation, temperature, and topography. Plant communities constitute one of the critical determinants influencing insect assemblages, with insect diversity demonstrating positive correlations with plant diversity (Fornoff et al. 2021). Enhanced vegetation canopy cover has been shown to promote increased insect diversity (Chen et al. 2009). Under global climate change scenarios, rising temperatures are driving poleward and upward elevational shifts in insect distributions (Chen et al. 2009), while humidity modulates insect diversity through its effects on developmental processes (Chang et al. 2008). Within optimal ranges, insect diversity increases with temperature and humidity (Beirão et al. 2020), whereas excessive thermal fluctuations reduce diversity (Moore et al. 2021). Furthermore, slope gradient significantly influences insect community distribution, with diversity increasing proportionally within moderate slope ranges (Marini et al. 2009).

### 4.3 Assembly Mechanisms of Heteropteran Communities

Quantifying the relative contributions of deterministic and stochastic processes to community assembly remains a central objective in ecological research (Beirão et al. 2020), as both processes are hypothesized to simultaneously govern community aggregation (Stegen et al. 2012). In this study, neutral community model (NCM) analyses indicated that deterministic processes predominantly controlled heteropteran community assembly across all elevational gradients—a pattern consistent with findings for beetle assemblages in low‐elevation regions (Zhang et al. 2018), but contrasting with prior studies on butterfly communities (Dini‐Andreote et al. 2015). This discrepancy in community assembly may be partially attributed to spatial scale dependency, habitat diversity, and distinct population dynamics (Gu et al. 2022). Current research suggests that insect communities are not only influenced by ecological factors at small scales but also by larger spatial scale effects due to the limited dispersal ability of insects (Ramzan et al. 2020). Ecological factors, such as temperature and topography, have a crucial impact on biodiversity, and different landforms can contribute to different species distribution patterns (Birkhofer et al. 2017).

Furthermore, MST analyses further demonstrated significant variations in the relative dominance of deterministic versus stochastic processes across elevational gradients in heteropteran communities. Our results revealed that communities at 1610–1860 m experienced the strongest stochastic processes, likely driven by anthropogenic disturbances (Li et al. 2022). This is similar to the findings of Miao et al. on the impact of climate and habitat changes on butterfly diversity (Hendrickx et al. 2009). This may be because, over longer time scales, some insects are less sensitive to changes in ecological factors and are more easily affected by habitat loss and alteration (Miao et al. 2020). Therefore, differences in the local insect species composition are best explained by a broader range of habitat categories.

Deterministic processes encompass abiotic and biotic factors that influence species presence/absence and relative abundance, which may contribute to community assembly processes at higher elevations (Pandit et al. 2009). Our findings demonstrate that deterministic processes dominated by heterogeneous selection exerted strong influences on the assembly of heteropteran communities distributed at 1360–1610 and 1860–2110 m, where environmental factors exhibited stronger associations with heterogeneous selection than biotic interactions. This provides robust evidence for the critical role of environmental filtering in relatively high‐elevation zones (Sun et al. 2018). However, these abiotic and biotic factors only partially explained variations in heteropteran community composition, aligning with numerous prior studies (Henriques et al. 2022). The unexplained variance in community assembly may be attributed to unmeasured abiotic factors, biotic interactions, and stochastic processes (Landi et al. 2018). Additionally, ecological responses to environmental changes may vary substantially among heteropteran species due to interspecific differences. The phytophagous heteropterans rely entirely on host plants and environmental niches; consequently, their limited environmental plasticity restricts their ability to adjust community composition in response to fluctuating conditions (Chen, Ren, et al. 2019; Liu et al. 2016). In this context, elucidating assembly mechanisms of heteropteran communities across elevational gradients is essential for interpreting and predicting their dynamics under multifaceted disturbances and may inform biodiversity conservation strategies amid environmental change.

### 4.4 The Effect of Complexity and Diversity on Stability Under Environmental Changes

Recent years have witnessed growing interest in how environmental changes alter the relationships among community complexity, diversity, and stability. However, most studies have focused on plant and microbial communities (Loreau and De Mazancourt 2013), with limited attention to insect communities, particularly heteropteran taxa. Habitat structure and environmental changes may affect insect gene flow and dispersal (Majeed et al. 2019). Studies on insect diversity in regional landscapes should examine the responses to changes in species richness and other metrics (Balakrishnan et al. 2014). This study's findings reveal that heteropteran community diversity plays a pivotal role in stabilizing heteropteran assemblages, serving as a buffer against the impacts of environmental fluctuations—a pattern consistent with the diversity‐stability hypothesis previously observed in plant and microbial communities (Shen et al. 2023).

Generally, communities with higher diversity exhibit greater stability over time. Because prolonged environmental changes induce pronounced fluctuations in species relative abundances, and the presence of more species with divergent responses to these changes enhances the probability of persistence under adverse conditions, ultimately improving community stability (Ives et al. 2000). Furthermore, due to increased niche differentiation and complementary resource use, communities harboring functionally significant trait variations among species are predicted to demonstrate heightened resilience to environmental perturbations (Cadotte et al. 2012). The hypothesis that complexity enhances stability constitutes a central ecological paradigm (Landi et al. 2018), increasingly substantiated through plant and microbial studies (Shen et al. 2022). Nevertheless, this mechanistic link warrants further empirical validation in this study. Our results demonstrate that both ecological variables and community diversity exert direct positive effects on stability. Beyond these immediate impacts, elevated community diversity may amplify community complexity, ultimately driving stability through cascading effects. Specifically, complexity mediates stability responses to environmental perturbations, as evidenced by concurrent significant shifts in relative abundances across taxonomic groups (Shen et al. 2022; Hernandez et al. 2021).

## 5 Conclusions

This study elucidates the diversity distribution patterns of heteropteran insects in the Daqing Mountain Nature Reserve and their differential responses to environmental drivers. SR and abundance of heteropteran exhibit elevational increases, with NDVI emerging as the primary driving factor. However, under multi‐factor interactions, heteropteran richness demonstrates an east‐to‐west declining gradient spatial pattern within the reserve, and deterministic processes dominate heteropteran community assembly across the reserve. Community diversity in heteropteran assemblages plays a critical buffer role in maintaining stability under environmental fluctuations. These insights substantially advance our understanding of heteropteran diversity dynamics under environmental change and their community assembly mechanisms, while providing actionable scientific foundations for conservation prioritization and management strategies. Nevertheless, the responses of insect diversity and the ecological service functions still require long‐term monitoring and assessment.

## Author Contributions

**Fuyuan Ta:** formal analysis (lead), software (lead), writing – original draft (lead). **Weina Wang:** data curation (equal), investigation (lead). **Hongru Yue:** formal analysis (supporting), investigation (equal). **Namuriga:** investigation (equal). **Husile:** investigation (equal). **Junlan Li:** investigation (equal), resources (equal). **Ning Wang:** conceptualization (equal), supervision (equal).

## Conflicts of Interest

The authors declare no conflicts of interest.

## Supporting information


**Table S1:** Backward stepwise regression method was applied to remove collinearity between variables with a variance inflation factor (VIF) less than 5, in order to maintain the best prediction accuracy for species richness.
**Table S2:** The blue‐shaded regions indicate the optimal parameter configurations, while the remaining data represent model parameters generated during the training process. These optimal configurations were subsequently employed to train the final model.
**Table S3:** Composition and proportion of Heteroptera in the Reserve.
**Table S4:** The sampling coverage was estimated using the “iNEXT” package in R across all grids. The SC value is used to assess whether sampling is sufficient to ensure an accurate description of species richness and ecosystems.
**Figure S1:** Species accumulation curves of the observed heteropteran samples in the Reserve.
**Figure S2:** PLS‐PM shows the soil, ecological variables, community diversity, and community complexity affecting heteropteran community stability.

## Data Availability

The data presented in this study are available upon request from the corresponding author. The data that support the findings of this study are available in the Supporting Information of this article.
