# Supplementary material for: Diversity Patterns and Assembly Mechanisms of Heteroptera Communities Under Environmental Change
Source: Ecol Evol. 2025 Sep 17;15(9):e72009. doi: 10.1002/ece3.72009 (PMC12441313; doi:10.1002/ece3.72009)
Supplement: Supplementary file 1 — Table S1: Backward stepwise regression method was applied to remove collinearity between variables with a variance inflation factor (VIF) less than 5, in order to maintain the best prediction accuracy for species richness. Table S2: The blue‐shaded regions indicate the optimal parameter configurations, while the remaining data represent model parameters generated during the training process. These optimal configurations were subsequently employed to train the final model. Table S3: Composition and proportion of Heteroptera in the Reserve. Table S4: The sampling coverage was estimated using the “iNEXT” package in R across all grids. The SC value is used to assess whether sampling is sufficient to ensure an accurate description of species richness and ecosystems. Figure S1: Species accumulation curves of the observed heteropteran samples in the Reserve. Figure S2: PLS‐PM shows the soil, ecological variables, community diversity, and community complexity affecting heteropteran community stability. [file ECE3-15-e72009-s001.docx]

**Supplementary Table S1.** Backward stepwise regression method was applied to remove colinearity be-tween variables with a variance inflation factor (VIF) less than 5, in order to maintain the best prediction accuracy for species richness.

| **Initial variables** | **Initial VIF** | **Final variables** | **Final VIF** |
| --- | --- | --- | --- |
| MAP | 252.701741 | MAT | 3.388913 |
| MAT | 216.840674 | NDVI | 3.067613 |
| NDVI | 3.837801 | pH | 2.027400 |
| pH | 185.571564 | Elevation | 4.042485 |
| Elevation | 9.775958 | SOC | 2.070683 |
| SOC | 2.419482 |  |  |
| TN | 194.14130 |  |  |

**Supplementary Table S2.** The blue-shaded regions indicate the optimal parameter configurations, while the remaining data represent model parameters generated during the training process. These optimal configurations were subsequently employed to train the final model.

| **mtry** | **min.node.size** | **RMSE** | **Rsquared** | **MAE** |
| --- | --- | --- | --- | --- |
| 2 | 4 | 0.5109720 | 0.3916137 | 0.4253365 |
| 2 | 5 | 0.5153420 | 0.3852608 | 0.4299271 |
| 2 | 6 | 0.5184438 | 0.3866804 | 0.4314506 |
| 2 | 7 | 0.5207103 | 0.3772681 | 0.4355378 |
| 2 | 8 | 0.5214751 | 0.3757305 | 0.4352315 |
| 2 | 9 | 0.5265161 | 0.3790302 | 0.4420289 |
| 2 | 10 | 0.5358279 | 0.3623378 | 0.4491663 |
| 3 | 4 | 0.5133340 | 0.3718871 | 0.4297099 |
| 3 | 5 | 0.5189884 | 0.3711962 | 0.4356399 |
| 3 | 6 | 0.5207487 | 0.3768682 | 0.4351414 |
| 3 | 7 | 0.5199294 | 0.3768110 | 0.4350509 |
| 3 | 8 | 0.5241986 | 0.3688036 | 0.4375750 |
| 3 | 9 | 0.5305315 | 0.3502386 | 0.4447633 |
| 3 | 10 | 0.5276105 | 0.3619744 | 0.4433422 |
| 4 | 4 | 0.5116290 | 0.3709594 | 0.4314986 |
| 4 | 5 | 0.5140609 | 0.3682462 | 0.4335920 |
| 4 | 6 | 0.5133692 | 0.3704379 | 0.4311077 |
| 4 | 7 | 0.5226144 | 0.3596936 | 0.4374529 |
| 4 | 8 | 0.5193767 | 0.3668339 | 0.4373284 |
| 4 | 9 | 0.5219466 | 0.3576007 | 0.4396940 |
| 4 | 10 | 0.5304727 | 0.3434525 | 0.4488395 |
| 5 | 4 | 0.5091957 | 0.3756370 | 0.4275859 |
| 5 | 5 | 0.5139609 | 0.3675439 | 0.4321131 |
| 5 | 6 | 0.5135394 | 0.3684997 | 0.4319034 |
| 5 | 7 | 0.5205175 | 0.3593431 | 0.4374115 |
| 5 | 8 | 0.5206374 | 0.3584565 | 0.4372098 |
| 5 | 9 | 0.5224500 | 0.3609817 | 0.4398669 |
| 5 | 10 | 0.5255901 | 0.3506845 | 0.4448010 |

**Supplementary Table S3.** Composition and proportion of Heteroptera in the Reserve.

| **Family** | **Genus** | | **Species** | | **Individual number** | |
| --- | --- | --- | --- | --- | --- | --- |
|  | **Number** | **Percentage (%)** | **Number** | **Percentage (%)** | **Number** | **Percentage (%)** |
| Acanthosomatidae | 3 | 2.91 | 7 | 4.4 | 357 | 4.01 |
| Alydinae | 2 | 1.94 | 6 | 3.77 | 63 | 0.71 |
| Anthocoridae | 2 | 1.94 | 4 | 2.52 | 247 | 2.77 |
| Berytidae | 2 | 1.94 | 3 | 1.89 | 53 | 0.59 |
| Blissidae | 1 | 0.97 | 1 | 0.63 | 6 | 0.07 |
| Coreidae | 3 | 2.91 | 4 | 2.52 | 21 | 0.24 |
| Cydnidae | 1 | 0.97 | 1 | 0.63 | 7 | 0.08 |
| Geocoridae | 2 | 1.94 | 4 | 2.52 | 46 | 0.52 |
| Gerridae | 3 | 2.91 | 5 | 3.14 | 190 | 2.13 |
| Lygaeidae | 6 | 5.83 | 8 | 5.03 | 2068 | 23.21 |
| Miridae | 6 | 5.83 | 7 | 4.4 | 963 | 10.81 |
| Nabidae | 5 | 4.85 | 7 | 4.4 | 1013 | 11.37 |
| Nepidae | 1 | 0.97 | 1 | 0.63 | 26 | 0.29 |
| Notonectidae | 1 | 0.97 | 1 | 0.63 | 1 | 0.01 |
| Oxycarenus | 4 | 3.88 | 5 | 3.14 | 237 | 2.66 |
| Pentatomidae | 18 | 17.48 | 28 | 17.61 | 1030 | 11.55 |
| Phymatidae | 1 | 0.97 | 1 | 0.63 | 39 | 0.44 |
| Pilnthisidae | 1 | 0.97 | 1 | 0.63 | 15 | 0.17 |
| Pyrrhocoridae | 1 | 0.97 | 1 | 0.63 | 10 | 0.11 |
| Reduviidae | 4 | 3.88 | 6 | 3.77 | 37 | 0.42 |
| Rhopalidae | 7 | 6.8 | 18 | 11.32 | 1121 | 12.58 |
| Rhyparochromidae | 14 | 13.59 | 16 | 10.06 | 841 | 9.44 |
| Saldidae | 3 | 2.91 | 4 | 2.52 | 238 | 2.67 |
| Scutelleridae | 3 | 2.91 | 4 | 2.52 | 96 | 1.08 |
| Tingidae | 7 | 6.8 | 13 | 8.18 | 40 | 0.45 |
| Urostylidae | 2 | 1.94 | 3 | 1.89 | 144 | 1.62 |
| Total | 103 | 100 | 159 | 100 | 8909 | 100 |

**Supplementary Table S4.** The sampling coverage was estimated using the “iNEXT” package in R across all grids. The SC value is used to assess whether sampling is sufficient to ensure an accurate description of species richness and ecosystems.

| **Assemblage** | **n** | **S.obs** | **SC** |
| --- | --- | --- | --- |
| A1 | 177 | 24 | 0.9549 |
| A2 | 73 | 15 | 0.9319 |
| A3 | 208 | 20 | 0.9713 |
| A4 | 127 | 22 | 0.9614 |
| A5 | 62 | 16 | 0.8877 |
| A6 | 99 | 19 | 0.9716 |
| A7 | 102 | 10 | 0.971 |
| A8 | 113 | 10 | 0.9915 |
| A9 | 45 | 6 | 0.9348 |
| A10 | 46 | 10 | 0.9584 |
| A11 | 204 | 24 | 0.966 |
| A12 | 226 | 14 | 0.9868 |
| A13 | 109 | 11 | 0.9727 |
| A14 | 255 | 21 | 0.9688 |
| A15 | 95 | 11 | 0.9792 |
| A16 | 16 | 4 | 0.8897 |
| A17 | 217 | 19 | 0.9956 |
| A18 | 16 | 6 | 0.9554 |
| A19 | 37 | 10 | 0.9245 |
| A20 | 37 | 12 | 0.8396 |
| A21 | 82 | 22 | 0.8914 |
| A22 | 67 | 9 | 0.957 |
| A23 | 189 | 23 | 0.9578 |
| A24 | 59 | 16 | 0.9361 |
| A25 | 88 | 18 | 0.921 |
| A26 | 88 | 18 | 0.9672 |
| A27 | 59 | 12 | 0.9503 |
| A28 | 49 | 7 | 0.899 |
| A29 | 109 | 11 | 0.9818 |
| A30 | 121 | 15 | 0.9588 |
| A31 | 15 | 9 | 0.6093 |
| A32 | 11 | 1 | 1 |
| A33 | 46 | 14 | 0.8715 |
| A34 | 29 | 13 | 0.7634 |
| A35 | 13 | 6 | 0.8681 |
| A36 | 25 | 3 | 1 |
| A37 | 70 | 16 | 0.9008 |
| A38 | 8 | 5 | 0.825 |
| A39 | 165 | 31 | 0.9336 |
| A40 | 160 | 27 | 0.9689 |
| A41 | 71 | 20 | 0.9026 |
| A42 | 77 | 11 | 0.9624 |
| A43 | 24 | 7 | 0.8818 |
| A44 | 37 | 8 | 0.9204 |
| A45 | 48 | 11 | 0.9384 |
| A46 | 75 | 18 | 0.9207 |
| A47 | 28 | 8 | 0.8967 |
| A48 | 20 | 8 | 0.9136 |
| A49 | 46 | 5 | 0.9792 |
| A50 | 36 | 12 | 0.9501 |
| A51 | 2 | 1 | 1 |
| A52 | 31 | 14 | 0.7805 |
| A53 | 29 | 5 | 1 |
| A54 | 26 | 9 | 0.8107 |
| A55 | 111 | 8 | 0.9821 |
| A56 | 125 | 22 | 0.8963 |
| A57 | 129 | 23 | 0.9465 |
| A58 | 135 | 28 | 0.9415 |
| A59 | 151 | 31 | 0.9211 |
| A60 | 69 | 13 | 0.9718 |
| A61 | 158 | 30 | 0.9248 |
| A62 | 166 | 32 | 0.9281 |
| A63 | 217 | 19 | 0.9771 |
| A64 | 151 | 14 | 0.967 |
| A65 | 105 | 25 | 0.8868 |
| A66 | 135 | 33 | 0.9046 |
| A67 | 136 | 26 | 0.9346 |
| A68 | 90 | 28 | 0.8906 |
| A69 | 152 | 16 | 0.9806 |
| A70 | 40 | 5 | 1 |
| A71 | 44 | 10 | 0.9575 |
| A72 | 70 | 21 | 0.8861 |
| A73 | 85 | 24 | 0.9073 |
| A74 | 114 | 23 | 0.9304 |
| A75 | 140 | 30 | 0.9079 |
| A76 | 52 | 16 | 0.8683 |
| A77 | 54 | 12 | 0.8896 |
| A78 | 21 | 8 | 0.8186 |
| A79 | 21 | 4 | 1 |
| A80 | 54 | 4 | 0.9822 |
| A81 | 42 | 5 | 1 |
| A82 | 7 | 1 | 1 |
| A83 | 75 | 9 | 0.9747 |
| A84 | 83 | 16 | 0.9527 |
| A85 | 136 | 17 | 0.9341 |
| A86 | 79 | 16 | 0.9117 |
| A87 | 16 | 10 | 0.641 |
| A88 | 88 | 2 | 1 |
| A89 | 31 | 7 | 0.9376 |
| A90 | 71 | 18 | 0.9026 |
| A91 | 29 | 15 | 0.8069 |
| A92 | 343 | 29 | 0.9768 |
| A93 | 34 | 14 | 0.8613 |
| A94 | 19 | 9 | 0.8579 |
| A95 | 31 | 7 | 0.9731 |
| A96 | 107 | 13 | 0.9541 |
| A97 | 7 | 4 | 0.6327 |
| A98 | 64 | 16 | 0.9243 |
| A99 | 14 | 5 | 0.801 |
| A100 | 26 | 9 | 0.8165 |
| A101 | 147 | 6 | 0.9934 |
| A102 | 18 | 3 | 0.9503 |
| A103 | 45 | 18 | 0.7817 |
| A104 | 19 | 5 | 0.8477 |
| A105 | 67 | 14 | 0.9407 |
| A106 | 36 | 11 | 0.8935 |
| A107 | 19 | 8 | 0.9053 |
| A108 | 66 | 21 | 0.8655 |
| A109 | 63 | 14 | 0.9216 |
| A110 | 135 | 23 | 0.9632 |


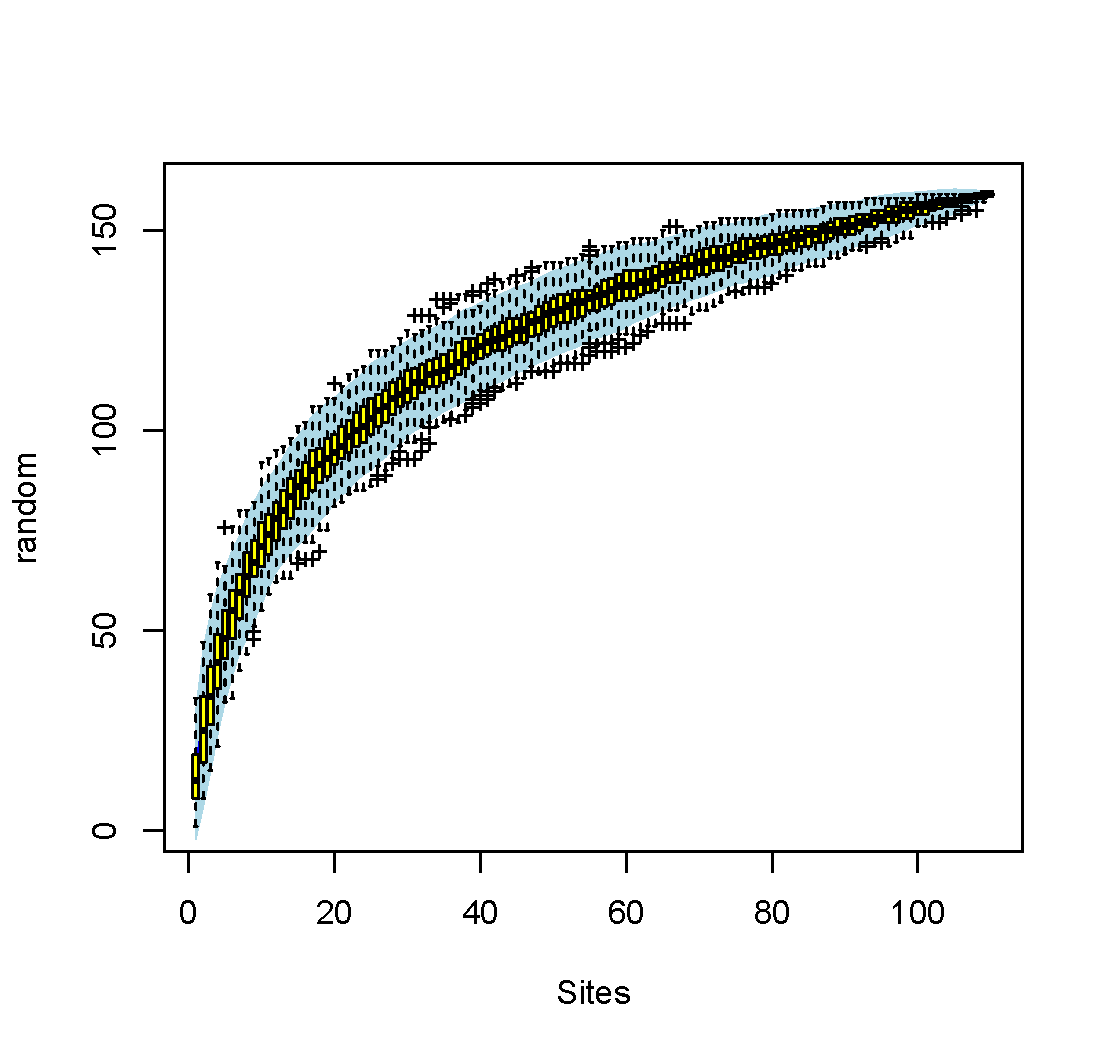


**Supplementary** **Figure S1.** Species accumulation curves of the observed heteropteran samples in the Reserve.


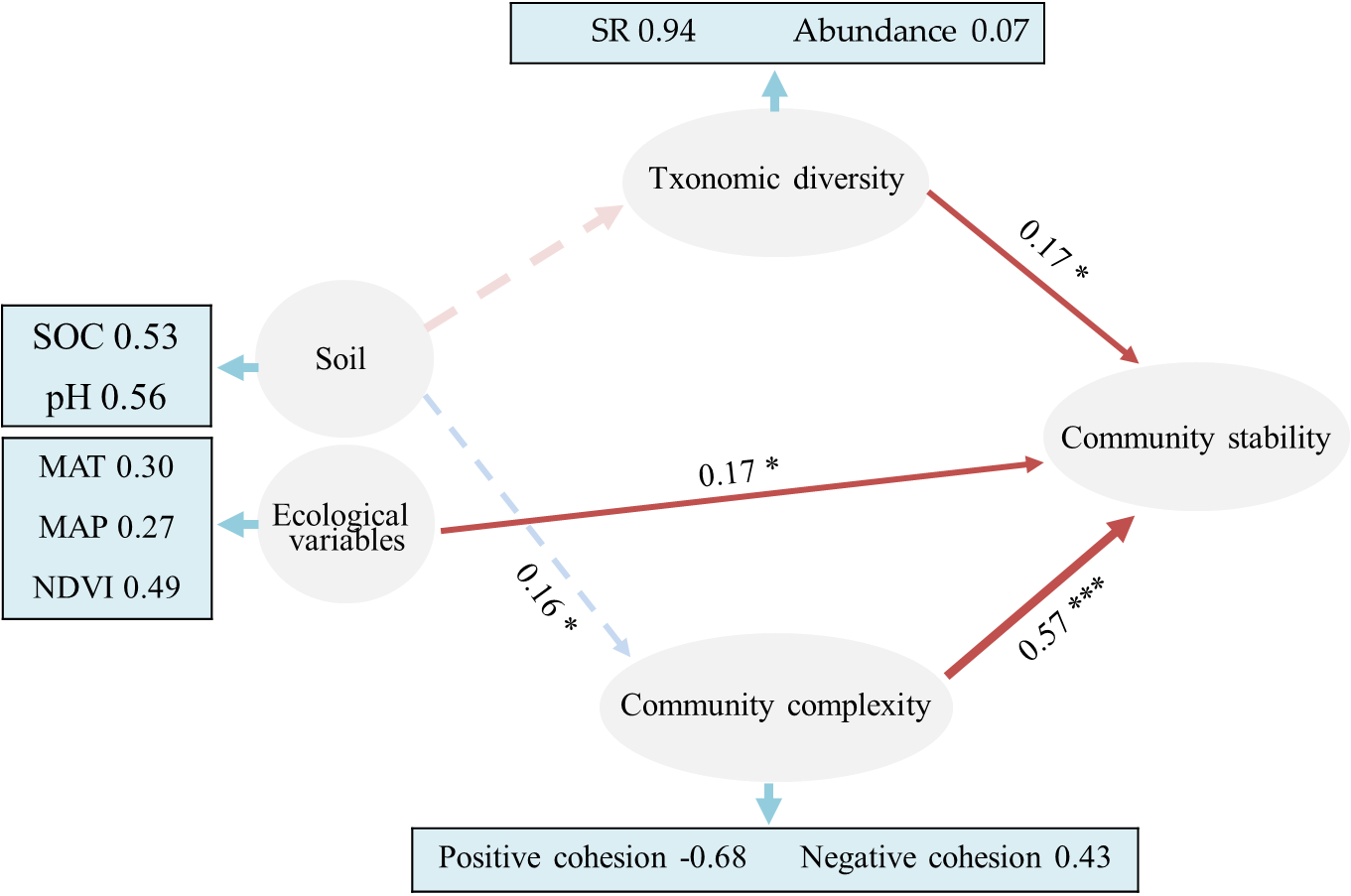


**Supplementary Figure** **S2.** PLS-PM shows the soil, ecological variables, taxonomic diversity, and community complexity affecting heteropteran community stability.
